# Rapid isolation and expansion of skin-derived precursor cells from human primary fibroblast cultures

**DOI:** 10.1242/bio.025130

**Published:** 2017-11-15

**Authors:** Leithe Budel, Karima Djabali

**Affiliations:** Epigenetics of Aging, Department of Dermatology, TUM school of Medicine, Technical University of Munich (TUM), 85748 Garching-Munich, Germany

**Keywords:** Skin-derived precursor cells, Adult stem cells, Acidic stress, Multipotent, Human fibroblast

## Abstract

Skin-derived precursor (SKP) cells have self-renewal and multipotent abilities and are found in the dermis. SKP cells have been isolated previously from pre-established dermal fibroblast cultures. In these procedures, long-term culture and low yield remain the crucial aspects requiring improvement. In this study, we exposed pre-established dermal fibroblasts to 30-min acid stress prior to isolating SKP cells (termed pH-SKP) and compared the yield to the previously published trypsin- and no-stress methods. Spheroid formation was confirmed and analyzed at days 3, 5 and 7*.* Stemness was investigated by immunohistochemistry for the stem cell markers Nestin, CD9, vimentin and NG2. Multipotency was investigated by differentiation into adipocytes, smooth muscle cells and fibroblasts. The pH-SKP spheroid yield at day 5 was four- and threefold higher than those obtained using trypsin- and no-stress methods, respectively. The expression of stem cell markers Nestin, CD9, vimentin and NG2 were significantly expressed in pH-SKPs compared to the fibroblast origin. Successful pH-SKP spheroid formation and differentiation were achieved and validated in 11 distinct human primary fibroblast lines. These results demonstrate that acute acidic stress treatment of dermal fibroblast cultures greatly improves SKP isolation, growth, yield and multipotency compared to previous methods.

## Abstract (precis)

**Summary:** Exposure of human dermal fibroblast cultures to a short period of acidic stress results in rapid isolation of skin-derived precursor (SKP) cells under the three-dimensional SKP culture conditions.

## INTRODUCTION

There is increasing interest in human skin-derived precursor (SKP) stem cells. These types of stem cells are multipotent and are present throughout adulthood (Fernandes et al., 2004; Toma et al., 2005, 2001). SKP cells reside within the dermis, express stem cell markers (Avilion et al., 2003; Lendahl et al., 1990; Mitsui et al., 2003; Pesce and Schöler, 2001), and can be readily isolated and expanded from skin biopsy, independent of the subject's age, site of biopsy, or disease (Joannides et al., 2004; Uchida et al., 2000). These factors, together with the ability of self-renewal (Toma et al., 2005), have made SKPs a topic of great interest as studies continue to find increasing potential in their multipotent ability.

Several studies have shown that SKPs can differentiate into various mesodermal and ectodermal cell types, such as smooth muscle cells, fibroblasts, neuronal cells, osteocytes, Schwann cells and adipocytes (Biernaskie et al., 2006; Hunt et al., 2008; Lavoie et al., 2009; Lebonvallet et al., 2012; Shu et al., 2014; Steinbach et al., 2011). Recently, SKPs were shown to differentiate into endodermal-like functional insulin-producing islet-like cells *in vitro*, suggesting that SKPs might even be a promising new tool for diabetes research (Bi et al., 2010; Mehrabi et al., 2015). Another study demonstrated the scalability of SKPs for large expansion and differentiation of Schwann cells (Walsh et al., 2016), which could be used in axon/nerve regeneration research (Kumar et al., 2016; Mozafari et al., 2015; Vasudeva et al., 2015) or as cell therapy for neurological injury [e.g. spinal cord injury (Yang et al., 2015)] or disease [e.g. multiple sclerosis (Willis and Fox, 2016)]. In addition to their multipotent ability, SKPs have shown promising results for stroke treatment because they secrete FGF and VEGF factors known to promote angiogenesis and stimulate neural stem cell proliferation (Mao et al., 2015). Consequently, SKPs are becoming attractive tools for disease modeling and therapy development because they are present during adulthood, are multipotent, secrete a wide range of factors and show regenerative potential.

The isolation of SKP cells from human skin biopsy has been well established (Fernandes et al., 2004; Toma et al., 2005, 2001). In general, this classic SKP isolation method requires the isolation of dermis, from which cells are cultured and expanded in a medium containing epidermal growth factor (EGF) and fibroblast growth factor 2 (FGF2), resulting in a suspension culture of SKP spheroids. The limitation of this method is the necessity of obtaining fresh skin biopsies for dermis tissue to isolate SKPs. This limitation increases the technical difficulty and decreases availability and accessibility for the study and use of SKPs, because access to human skin samples is limited and requires ethical committee approval. In addition, large-scale expansion of SKP cultures rapidly increases cost due to the addition of required growth factors to the culture medium.

To circumvent the limitations inherent to the classic SKP isolation, we previously established a method for SKP isolation from pre-established dermal primary fibroblast cultures (Wenzel et al., 2012a). In this method, trypsin is used as a temporary cellular stressor on fibroblasts, followed by a 21-day culture in conventional SKP medium (Wenzel et al., 2012b). In a study by Hill et al. (2012), similar results for SKP cell isolation from pre-established fibroblasts were reported without the application of stress. The use of primary fibroblast cultures provides certain advantages over the classic method because they are easily obtained from numerous cell banks (Coriell and ATCC Biorepositories) for normal and disease conditions, do not require growth factors for expansion, and can be efficiently stored for decades. Therefore, pre-established primary fibroblast cultures represent an attractive alternative to skin as a source for SKP isolation.

Only the two studies cited above (Hill et al., 2012; Wenzel et al., 2012b) have reported the alternative isolation of SKP cells from primary fibroblast cultures. Both methods remain subject to certain limitations such as the reduced multipotency of SKPs isolated from fibroblast cultures at high passage number, the requirement for long-term SKP culture, and low yield. These limitations must be addressed to provide either improved methods or alternative approaches for SKP isolations.

Based on previous observations that acidic pH affects certain cellular properties such as proliferation (Fitzgerald et al., 1997), we hypothesized that temporary acidic stress on primary fibroblast cultures would improve and accelerate the isolation and growth of SKP cells *in vitro*. We therefore designed a method in which exposure of a fibroblast preparation to an acute acidic stress environment results in SKP isolation, with strong spheroid growth within 5 days under three-dimensional SKP culture conditions. These SKP spheroids exhibit multipotent ability independent of the fibroblast line of origin or passage number *in vitro*. This novel strategy provides an improved alternative method for SKP isolation from pre-established primary fibroblasts.

## RESULTS

To test our hypothesis that acute acidic stress on pre-established primary fibroblast monocultures would permit the rapid isolation and expansion of SKP cells, we established an acidic stress isolation method and compared this protocol with the previously published trypsin-based isolation method (Wenzel et al., 2012b) and no-stress method (Hill et al., 2012) by examining SKP growth. The stemness of the SKP spheroids isolated after acid stress was then further investigated.

### Defining the acidic pH stress for the SKP isolation method

To determine the optimal acidic stress pH for our method, we first investigated the effect of low acidic stress on primary dermal fibroblasts using a pH range from 7 to 5 in Hank's balanced salt solution (HBSS). Normal HBSS (pH 7.4) was used as a control, and pH 2.5 was used to induce cell death (Fig. S1). Fibroblasts were incubated in suspension for 30 min in HBSS buffer with varying pH at 37°C with 5% CO_2_ and were then transferred after removal of the HBSS buffer into SKP classic culture medium (see Materials and Methods). After treatment, cell viability was determined by fluorescence-activated cell sorting (FACS). The viability of normal HBSS-treated fibroblasts showed that the direct effect of the experimental conditions resulted in a total cell death of ∼4.5% at 0 h post-treatment to ∼20% at 2 h, with no further reduction at 20 h post-treatment (Fig. S1). Fibroblast suspensions exposed to pH 7.0 and below showed high levels of cell death after 20 h post-treatment (Fig. S1). The percentage of cell death between pH 6.7 and pH 5.3 reached a plateau at ∼34% but increased significantly further at pH 5.0 (∼45% cell death) (Fig. S1). These findings indicated that an average of 66% of the fibroblast suspension remained viable at 20 h post-treatment between pH 6.7 and pH 5.3. To assess the effect of acidic pH stress on SKP isolation from primary fibroblast cultures, we selected pH 5.7.

### Acidic stress treatment on primary dermal fibroblasts induces rapid spheroid formation and growth

**Fig. 1.**
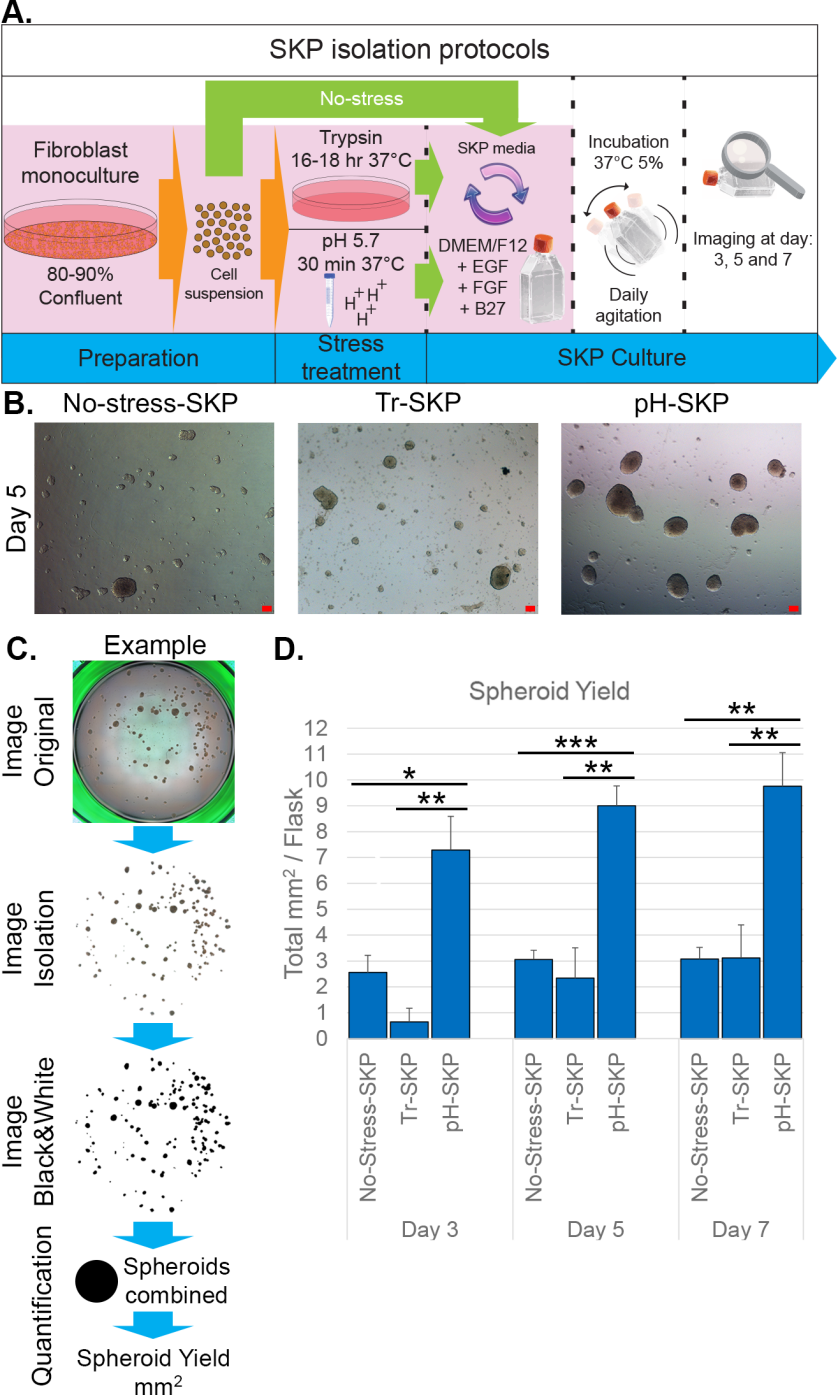
**Acute exposure of primary fibroblast to pH 5.7 induces rapid SKP spheroid formation and growth.** (A) Schematic representation of methods of SKP isolation from primary fibroblasts using no stress, trypsin stress (Tr-SKP) or acidic stress (pH-SKP). (B) Representative images of SKP spheroid cultures at day 5 using the no-stress, trypsin-stress or low pH method are shown (*n*=6). Scale bar: 100 µm. (C) The panel outlines the spheroid quantification process starting from a whole-well image of a total SKP culture. Briefly, spheroids are isolated from the image original, and black color is then overlaid for binarization. All spheroid numbers and sizes are combined to calculate the spheroid yield per culture. (D) Graph represents the spheroid yield at days 3, 5 and 7 in mm^2^. Data are expressed as the mean±s.d. (***P<0.005, **P<0.01, *P<0.05; Student's *t*-test; *n*=3).

To assess the effect of low acidity on SKP isolation, a 30-min acidic stress treatment of pH 5.7 in HBSS buffer at 37°C was used on pre-established dermal primary fibroblasts derived from foreskin. These cultures were compared to cultures subjected to no-stress or trypsin-stress conditions as described previously [Materials and Methods and Wenzel et al. (2012b)]. After treatment, all cell groups were cultured in SKP medium and termed no stress-SKP, Tr-SKP (trypsin-based) and pH-SKP (low pH-based) (Fig. 1A).

Images of all SKP cultures were acquired at various days to monitor spheroid formation and growth. Clear spheroid formation in pH-SKP cultures was detected by day 3, and further analysis showed that 5 days was sufficient for pH-SKP to form numerous large spheroids (Fig. 1B). By contrast, the no-stress and trypsin-based methods produced fewer spheroids and required longer culture periods of up to 21 days to form numerous spheroids, consistent with previous reports (Hill et al., 2012; Wenzel et al., 2012b). These differences indicated that spheroid development under pH-SKP isolation conditions was significantly faster than under the other isolation conditions.

To further determine the effect of low pH treatment on global spheroid culture, the spheroid number and size were quantified for all conditions. The entire cultures were collected in 48-wells plates, and images of entire wells were acquired as described in the Materials and Methods (Fig. 1C). The spheroids were first digitally isolated from the original image, followed by black-and-white adjustment for binarization and quantification of each spheroid by ImageJ (Fig. 1C). The numbers and sizes of the spheroids were combined to give the total spheroid yield per culture and condition (Fig. 1D). The total spheroid yield was much higher using pH-SKP conditions compared to no-stress or Tr-SKP cultures (Fig. 1D). Moreover, a clear difference was already apparent by day 3. Importantly, when typical large spheroids were detected by day 5, the pH-SKP spheroid yield was three- to fourfold higher than those observed under no-stress and trypsin-stress conditions, respectively (Fig. 1D). Collectively, these findings indicate that exposure of primary fibroblast cells to acute acidic stress (pH 5.7) induces more efficient spheroid formation and growth than previously reported methods.

### pH-SKP spheroids express multipotent stem cell markers

**Fig. 2.**
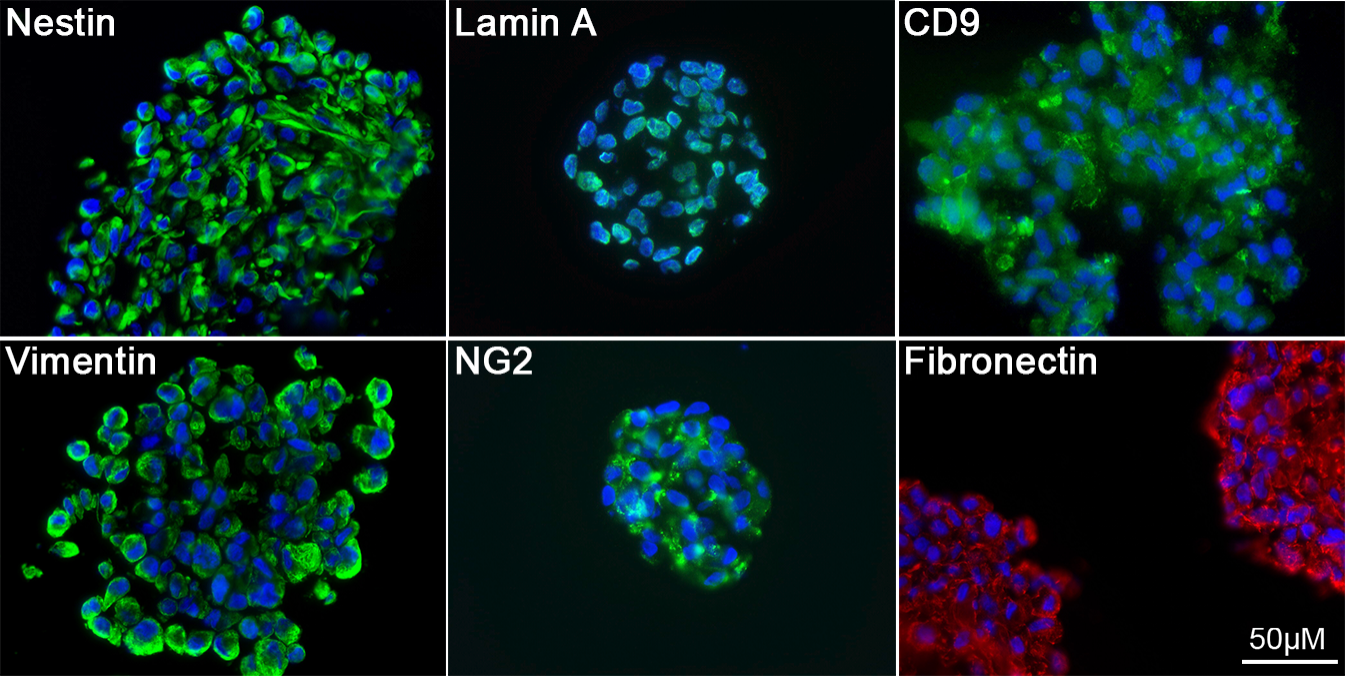
**Day 5 pH-SKP cells express the neuronal crest and multipotent stem cell markers.** Immunohistochemistry of the indicated stem cell markers was performed on day 5 pH-SKP spheroids cryo-sections (*n*=6). Representative images of pH-SKP spheroids derived from primary fibroblast GM05565 at passage 12 are shown. The nuclear lamina marker lamin A, vimentin and fibronectin were labeled as well. Sections were counterstained for DNA with DAPI (blue). Scale bar: 50 µm.

To determine the stemness of pH-SKP spheroid cells, the expression of stem cell markers was evaluated by immunohistochemistry of pH-SKP spheroid cryo-sections (Fig. 2). Sections were positive for the neuronal crest marker Nestin (Lendahl et al., 1990) and the multipotent marker CD9 (International Stem Cell Initiative, 2007) (Fig. 2). Furthermore, neuron-glial antigen 2 (NG2), also known as melanoma chondroitin sulfate proteoglycan (MCSP), a marker of glial precursors (Stegmüller et al., 2002) and epidermal stem cells (Legg et al., 2003), was positively labeled in pH-SKP spheroids (Fig. 2). In addition to stem cell markers, positive labeling was observed for lamin A, vimentin and fibronectin, showing that pH-SKP spheroids also express proteins that are present in fibroblast cells. Collectively, these results show that pH-SKP spheroids at day 5 in culture expressed stem cell markers indicating the stemness properties of these pH-SKP cells.

### pH-SKPs can be isolated from human primary fibroblast cultures derived from all ages, body sites and passage numbers

**Fig. 3.**
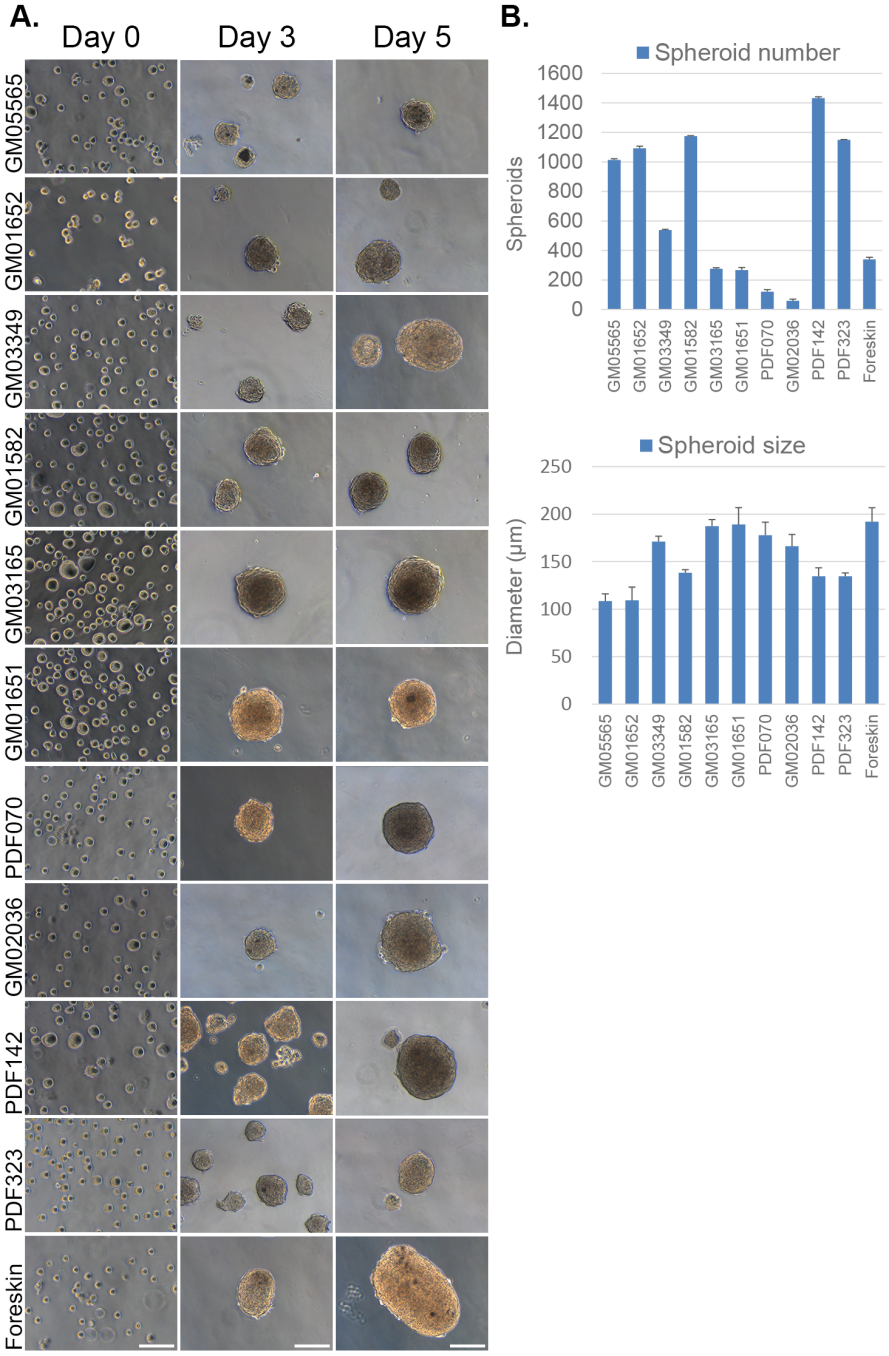
**Efficient isolation of pH-SKP spheroids from eleven primary fibroblast lines.** (A) Images show representative 5-day growth overview of pH-SKP spheroids from the eleven primary fibroblast lines at days 0, 3 and 5 in SKP medium (*n*=3). Scale bar: 100 µm. (B) Graph illustrates day-5 measurements of pH-SKP spheroids for each cell line representing in upper panel: spheroid number (*n*=3), and lower panel: spheroid size in diameter based on 50 random spheroid measurements (*n*=5). Data are expressed as the mean±s.d.

To further investigate the potential of the acidic stress method for SKP isolation, various primary fibroblast lines were used for proof of concept. In total, eleven primary fibroblast lines from passage numbers between 7 and 16 and originating from various body sites including the inguinal area, arm and foreskin were used to isolate pH-SKPs (Table 1). After acidic treatment, cells were cultured in suspension for 5 days in SKP medium. On days 3 and 5, successful formation of spheroids was observed for all cell lines (Fig. 3A).

**Table 1.**
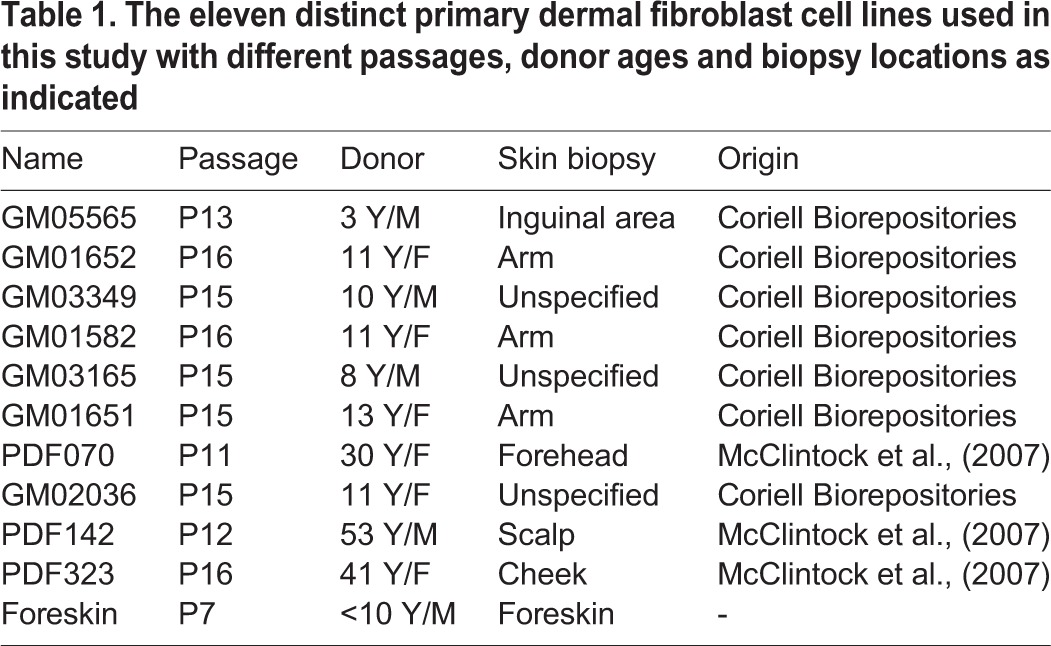
**The eleven distinct primary dermal fibroblast cell lines used in this study with different passages, donor ages and biopsy locations as indicated**

Further measurements of the spheroids revealed several differences among cell lines. The spheroid number showed the largest variance, with an average of 678 spheroids among all cell lines and a standard deviation of 490, which represents a 72% variance (Fig. 3B, spheroid number). These results indicate that the number of spheroids obtained during isolation is dependent on the starting material but has no clear correlation with skin biopsy site, passage number or donor age, suggesting that the number of spheroids formed is cell line- and experiment-dependent. However, the variance in the measured spheroid size among cell lines was lower. An average diameter of 155 µm with a standard deviation of 30 µm, was observed, representing a variance in spheroid size of only 19% among all cell lines (Fig. 3B, spheroid size).

Collectively, these findings indicate that acidic stress treatment at pH 5.7 successfully induced SKP isolation from primary fibroblast cultures independent of donor age, skin biopsy site or passage number. To further characterize the multipotent properties of these pH-SKP spheroids, we evaluated their differentiation potential.

### pH-SKP cells differentiate into adipocytes

**Fig. 4.**
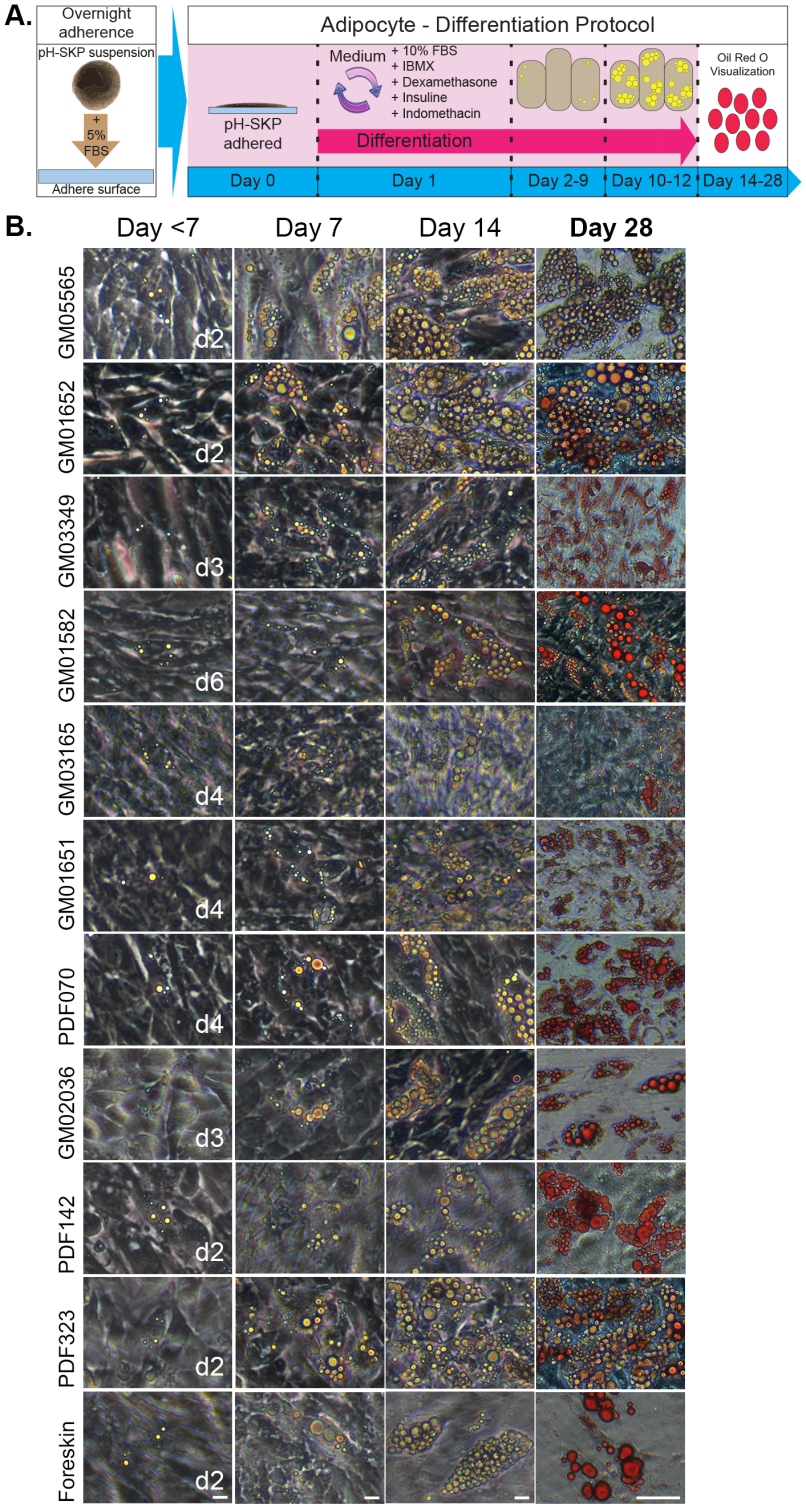
**pH-SKP spheroids can differentiate into adipocytes.** (A) Schematic overview outlines the pH-SKP adipocyte differentiation protocol. Briefly, pH-SKP spheroids were adhered overnight in SKP medium supplemented with 5% FBS, followed by (day 0) incubation in adipocyte differentiation medium for 14 to 28 days. Then, Oil Red O (ORO) staining was performed. (B) ORO staining was performed on the indicated days on pH-SKPs differentiated to adipocyte differentiation. Scale bar: first to third column, 10 µm; fourth column, 50 µm (*n*=3).

To assess the multipotent potential of pH-SKP cells, adipocyte differentiation was induced as outlined in the schematic representation of the adipocyte differentiation protocol (Fig. 4A). Briefly, pH-SKP spheroids at day 5 were first allowed to adhere in 5% fetal bovine serum (FBS)-supplemented SKP medium for a period of 16 to 18 h. Then, the medium was replaced with differentiation medium containing adipocyte-inducing factors (10% FBS, IBMX, dexamethasone, insulin and indomethacin as indicated in the Materials and Methods). This time point was considered day 0 of adipocyte induction. Lipid vesicle formation was monitored and visualized by Oil Red O (ORO) staining between days 14 and 28.

At day 5, pH-SKP spheroids from eleven distinct primary fibroblast cell lines (Table 1) were directed to differentiate into adipocytes for a total period of 28 days (Fig. 4B). The formation of lipid triglycerides in a range of 3 to 8 small lipid vesicles could generally be detected at 2 days post-induction by standard bright field microscopy (Fig. 4B, first column). pH-SKP cells derived from all cell lines from passage 7 to 16 showed lipid formation, with the earliest detection at day 2 and latest at day 6 post-induction. In addition, SKPs derived from passage 21 fibroblast cultures were competent to differentiate into adipocytes (Fig. S2). From days 7 to 28 in adipocyte differentiation medium, cells from all cultures showed increasing numbers of lipid droplets of increasing size (Fig. 4B).

**Fig. 5.**
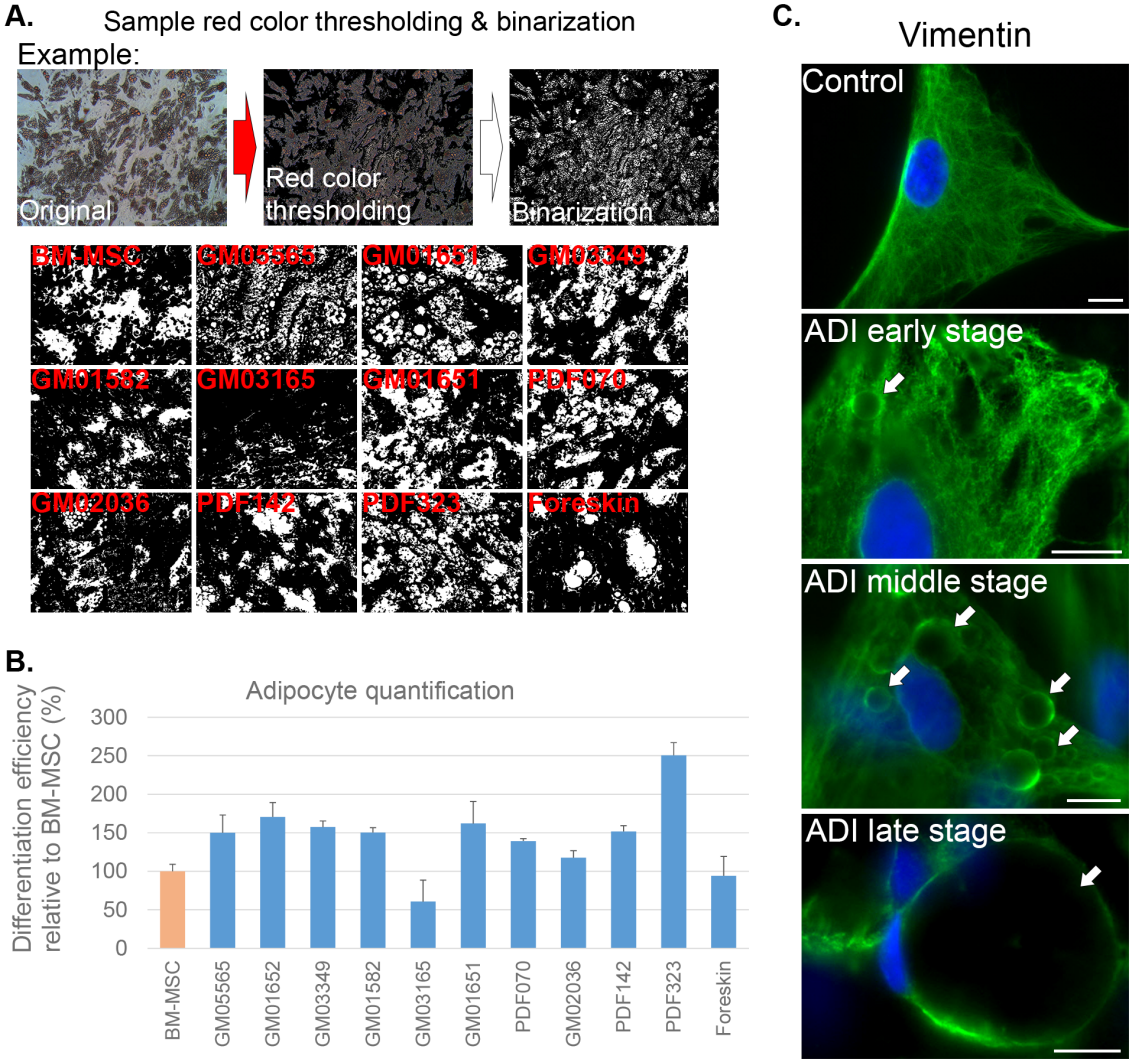
**pH-SKPs efficiently differentiated** **into adipocytes and show vimentin network remodeling.** (A) Oil Red O (ORO) color threshold analysis of adipocyte-differentiated pH-SKPs isolated from eleven different fibroblast cell lines and control BM-MSC. The top panel shows an example of the methodology of color thresholding, with initial isolation of red color followed by binarization, where white represents stained lipids. The bottom panel shows a representation of the indicated cell line at day 28 of adipocyte differentiation after digital processing. (B) A graph illustrating the red color analysis, representing an estimate of the lipid percentage in a field of 1317 ×1317 µm per cell line; data are normalized to the BM-MSC and expressed as the mean±s.d. (*n*=3). (C) Representation of vimentin status during the development of adipocytes. Control fibroblasts and early-, middle- and late-stage adipocytes were immunolabeled for vimentin (green) and counterstained for DNA with DAPI (blue); the arrows indicate vimentin-encaged lipids. Scale bar: 10 µm. ADI, adipocyte.

To quantify the adipocyte differentiation potential of each culture, the lipid presence was estimated per cell line via digital image analysis. A simplified image analysis method based on the report of Deutsch et al. (2014) was applied. Here, via red color thresholding, stained cellular lipids were digitally isolated, which was then followed by binarization of the image to make it suitable for digital analysis (Fig. 5A). Raw images for digital ORO analysis were obtained by imaging three different locations with the highest lipid density per cell line with a 10× objective, resulting in a total acquired image field of 879 µm×658 µm of which the color percentage was determined (Fig. 5B). The values of bone marrow mesenchymal stem cell (BM-MSC)-derived adipocytes served as a positive control and were set as 100% (Fig. 5A). Overall, pH-SKP samples showed a higher tendency to differentiate into adipocyte relative to BM-MSC (Fig. 5B). Beside GM03165 and foreskin pH-SKP samples, all the other samples exhibited an average of 146% adipocyte differentiation relative to BM-MSC (Fig. 5B). Hence, we tested whether SKPs isolated with no-stress (NS-SKPs) or fibroblast cultures could differentiate into adipocytes (Fig. S3). Fig. S3A indicate that fibroblast cultures directly exposed to adipocyte differentiation media showed very rare cells containing lipid droplets at day 21. Whereas numerous cells exhibited large lipid vesicles in adipocytes derived from NS-SKP and pH-SKP (Fig. S3A). Digital determination of the percentage of adipocytes present in the different samples showed that only 13% of the fibroblasts were able to differentiate into adipocytes (Fig. S3B). In contrast, 112% of the NS-SKP and 141% of the pH-SKP preparations were capable of adipocyte differentiation relative to BM-MSC (Fig. S3B). These results demonstrate that SKPs were competent to differentiate into adipocytes independently of the method of isolation. Furthermore, the differentiation of rare adipocytes in the fibroblast cultures indicate that those few cells might most likely originate from the pre-existing SKPs in the fibroblast cultures.

Next, vimentin, a type III intermediate filament protein, was analyzed to follow the reorganization of the cytoskeleton in pH-SKP cells at different stages of adipocyte differentiation as previously reported (Franke et al., 1987; Verstraeten et al., 2011). Adipocytes were immunolabeled for vimentin at day 28 of differentiation; at which point early-, middle- and late-stage adipocytes were present because not all cells from an adhered spheroid initiated differentiation simultaneously (Fig. 5C). At the early stages of adipocyte differentiation minor re-organization of the vimentin filaments was observed, enveloping the lipids with a spherical caged structure (Fig. 5C). At the middle stages of differentiation, cells showed further re-organization of the vimentin filaments, as indicated by increased vimentin-positive sphere-like formation in the cytoplasm. At later stages, large lipid droplets formed and were totally wrapped with vimentin filaments (Fig. 5C).

These findings indicate that pH-SKP spheroids isolated from primary fibroblasts have the ability to re-organize their vimentin network around the developing lipid droplets upon the induction of adipocyte differentiation. These results further support the multipotency and plasticity of pH-SKP cells.

### pH-SKP cells can differentiate into smooth muscle and fibroblast cells

**Fig. 6.**
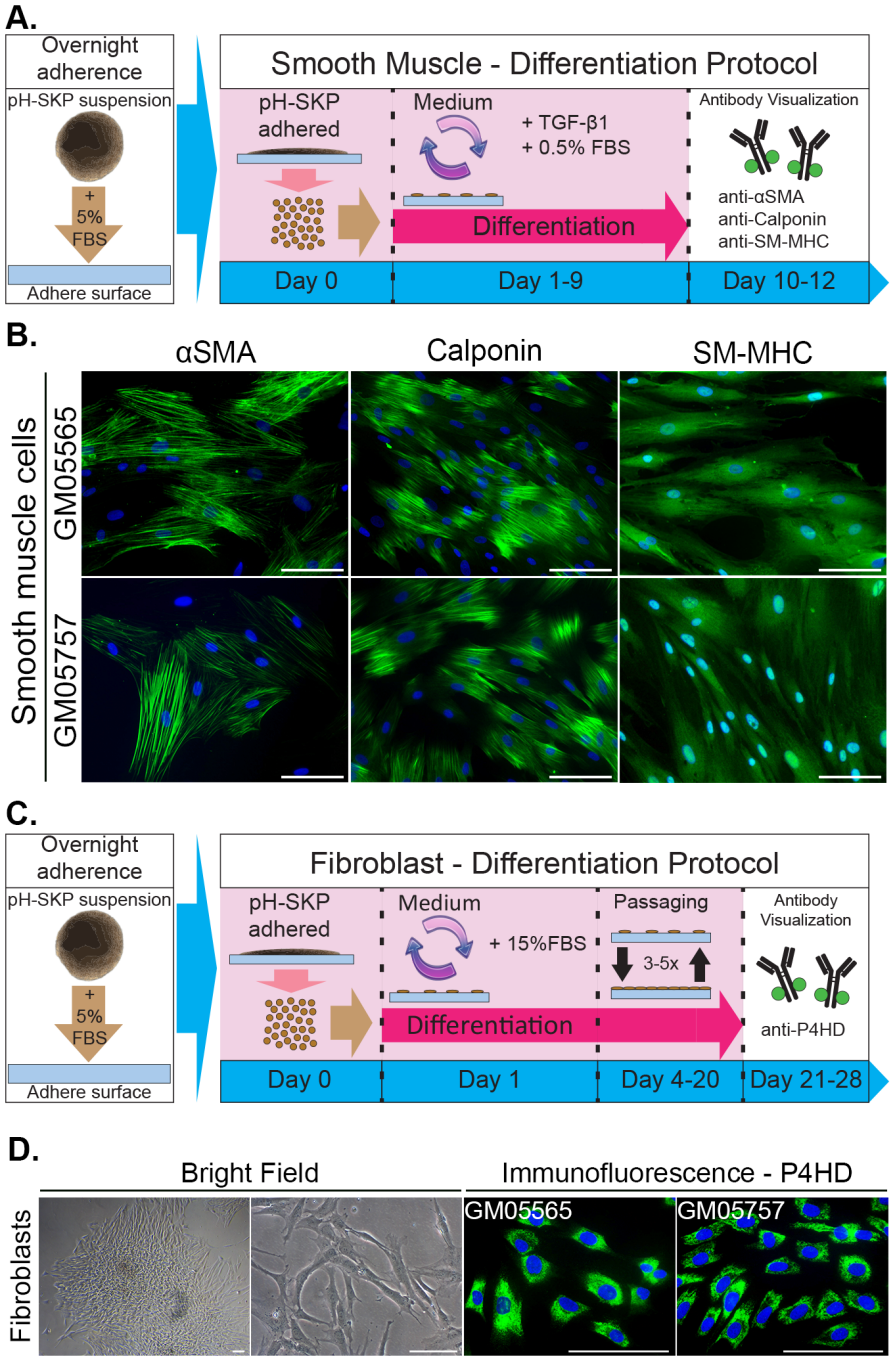
**pH-SKP cells can differentiate into smooth muscle cells and fibroblasts.** (A) Schematic overview of the pH-SKP SMC differentiation protocol. Briefly, pH-SKP spheroids were first adhered for 16-18 h in SKP medium supplemented with 5% FBS. The spheroids were then dissociated and re-seeded at low density, followed by SMC differentiation induction in SMC medium for 10 to 12 days. (B) SMC differentiation was assessed by immunolabeling of αSMA, calponin and SM-MHC as indicated. Representative images of SMCs derived from pH-SKP cells isolated from GM05565 and GM05757 at day 10 are shown. DNA was counterstained with DAPI (blue). Scale bar: 100 µm. (C) Schematic overview of the pH-SKP fibroblast differentiation protocol. Briefly, after adherence and low-density re-seeding, pH-SKP cells were cultured in 15% FBS-supplemented medium followed by 3-5 passages. Cells were immunolabeled for the fibroblast marker P4HD. (D) Representative images of fibroblasts differentiated from pH-SKP cells isolated from GM05565 and GM05757 fibroblast lines are shown. On the left, the bright field shows the morphology of an adhered pH-SKP spheroid and the fibroblast morphology of pH-SKP cells directed to differentiate into fibroblast cell type at passage 4. The right images are fibroblasts derived from pH-SKP differentiation labeled for P4HD (green) and counterstained with DAPI for DNA (blue). Scale bar: 100 µm. (*n*=3).

To further assess the multipotent potential of pH-SKP cells, smooth muscle and fibroblast differentiation were induced. A schematic representation of the smooth muscle cell differentiation protocol is shown in Fig. 6A. Briefly, pH-SKP spheroids at day 5 were allowed to adhere and induced to differentiate as described in the Materials and Methods. Smooth muscle cells (SMCs) derived from pH-SKP cells were fixed between days 10 and 12 post-induction and immunolabeled for smooth muscle α-actin (αSMA), calponin and smooth muscle myosin heavy chain (SM-MHC), proteins that are typically expressed in SMCs (Rensen et al., 2007). All SMC markers were expressed in SMCs derived from pH-SKPs at day 10 under SMC differentiation conditions (Fig. 6B). We also evaluated the potency of SMC differentiation of NS-SKPs and fibroblast cultures relative to pH-SKPs (Fig. S4A). The results showed that all SMC markers (aSMA, calponin and SM-MHC) were absent at day 0 of SMC differentiation, but were detected at day 11 (Fig. S4A). However, the differentiation efficiency was very variable with only 15% SMCs detected in the fibroblasts, 29% in NS-SKPs and 76% in pH-SKPs cultures (Fig. S4B). These results indicate that pH-SKPs were more competent to differentiate into SMCs compared to NS-SKPs (Fig. S4B). Only few cells in the fibroblast cultures showed potency to differentiate into SMCs. These findings indicate that the SMCs induced in the primary fibroblast cultures might originate from the pre-existing SKPs present in those cultures.

In addition to SMC differentiation, pH-SKP cells were also able to differentiate into fibroblast cells. A schematic representation of the fibroblast differentiation protocol is shown in Fig. 6C. Briefly, after adherence, pH-SKP cells were cultured in fibroblast culture medium as described in the Materials and Methods. After 3 to 5 passages, the morphology of differentiated pH-SKP cells was similar to that of normal fibroblasts (Fig. 6D). Moreover, fibroblasts derived from pH-SKPs were positive for the fibroblast marker prolyl-4-hydroxylase beta (P4HB), an enzyme involved in collagen synthesis localized in the endoplasmic reticulum (Myllyharju, 2003) (Fig. 6D).

In summary, our findings indicate that a 30-min exposure of primary fibroblast cultures to pH 5.7 induces rapid cell proliferation and spheroid development when grown in SKP medium compared to previously reported methods (Hill et al., 2012; Wenzel et al., 2012b). The generation of pH-SKP spheroids occurred faster, showed clonal expansion ability (Fig. S5) and provided fourfold higher yield compared with previous procedures, obviating long-term culture prior to use. Moreover, the pH-SKPs expressed typical multipotent stem cell markers and showed potency to differentiate into adipocytes, smooth muscle cells and fibroblasts *in vitro*. Additionally, pH-SKP isolation, expansion and differentiation were replicated using various primary dermal fibroblast lines independent of the donor age, biopsy site or passage number *in vitro*.

## DISCUSSION

Current isolation methods of SKP cells are thwarted by certain limitations. The classic isolation method introduced by Toma et al. (2005) requires fresh skin tissue and long culture times to isolate SKPs. A second strategy for SKP isolation (Hill et al., 2012; Wenzel et al., 2012b) circumvents the need for fresh skin tissue by using pre-established dermal fibroblast cultures available from commercial cell banks. However, limitations such as long-term culture, low yield and requirement of early cell passage reduce the utility of this method, highlighting the need for further improved isolation strategies.

In this study, we present a novel SKP isolation procedure as an alternative that overcomes the limitations of previous methods. We report that exposure of human primary dermal fibroblast cultures to acidic stress (pH 5.7) for 30 min at 37°C induces rapid spheroid formation and expansion of SKP cells, termed pH-SKPs within 5 days *in vitro*. These pH-SKPs express stem cell markers such as Nestin, CD9, vimentin and NG2 and exhibit multipotent properties. Moreover, pH-SKPs showed higher ability to differentiate into smooth muscle cells (SMC) and adipocytes compared to SKPs isolated with the no stress method. No derivation of neuronal lineage was attempted and remains to be investigated in the future. Additionally, we found that fibroblast cultures directly exposed to SMC or adipocyte media exhibited a few differentiated cells. We propose that these rare cells that are present in the primary fibroblast cultures might be the pre-existing SKPs.

pH has long been known to influence cellular functions such as cell growth and protein synthesis (Mackenzie et al., 1961). Regulation of the intracellular pH (pHi) is therefore crucial to maintain cell function. One regulator of pHi is the sodium-hydrogen exchanger (NHE), which removes intracellular acid via exchange of a proton for an extracellular sodium ion (Aickin and Thomas, 1977). The first isoform discovered, NHE1 is located in the plasma membrane (Kemp et al., 2008; Slepkov and Fliegel, 2002). When the pHi drops below a certain threshold level, the NHE1 protein is directly activated via an internal allosteric proton-binding regulatory site. In turn, NHE1 affects various cell functions such as cell survival, proliferation (Kapus et al., 1994), and cytoskeleton (Denker et al., 2000) and actin filament assembly (Meima et al., 2009). Interestingly, we found that short exposure to acidic extracellular pH (pHe) induced rapid proliferation of SKPs present in the fibroblast culture of origin. Although outside the scope of the present study, further studies are needed to fully characterize this process, but we suggest here that NHE1 activation might play a significant role. More detailed investigations on the mechanisms underlying the signaling pathways elicited by acidic pH stress will undoubtedly offer new options for further improvements of SKP isolation and possibly other adult stem cell populations present in tissues other than the skin.

The rapid expansion of pH-SKPs might hold great potential for clinical or translational purposes. For example, pH-SKP differentiation into functional insulin-producing islet-like cells could be applied for the development of future diabetes therapy (Mehrabi et al., 2015). Another area to be further investigated is the utilization of pH-SKP cells in drug and disease modeling. For example, SKP cells can be isolated from the skin of patients suffering from diseases such as Hutchinson Gilford Progeria Syndrome (HGPS) (Hill et al., 2012; Wenzel et al., 2012b) and Hirschsprung's disease (HSCR) (Kwok et al., 2013). Using fibroblast cultures for expansion and storage would obviate the need for repeated skin biopsies, which significantly elevate patient stress. Moreover, for basic science and other applications, fibroblasts can be easily purchased from commercial biorepositories and used for pH-SKP isolation and differentiation into various cell types without the need for surgical interventions.

In conclusion, we report an efficient method for rapid SKP cell isolation using a short acidic stress treatment of pH 5.7 on primary dermal fibroblasts. Ready-to-use pH-SKP preparations were obtained four times faster compared with previously reported methods and exhibited typical stem cell markers and differentiation potencies. Furthermore, pH-SKPs can be isolated from fibroblast preparations derived from all ages, body sites and even late culture passages. In all cases, the isolated pH-SKPs showed similar spheroid expansion, morphology, stemness markers and differentiation potentials within 5 days *in vitro*. Thus, this pH-SKP isolation method is an efficient, rapid and standardized method to generate large numbers of SKPs. We acknowledge that the potential use for *in vivo* applications will require further *in vivo* studies to determine their safeties, survival, function and differentiation. Nevertheless, pH-SKPs can become valuable tools to basic and translational research and possibly one day in regenerative medicine.

## MATERIALS AND METHODS

### Cell culture

The human primary dermal fibroblast lines GM05565, GM05757, GM01652, GM03349, GM01582, GM03165, GM01651 and GM02036 were all obtained from Coriell Biorepositories (New Jersey, USA); PDF070, PDF142 and PDF323 were obtained from a previous study (McClintock et al., 2007). The above cell lines were all established from skin biopsies of unaffected individuals (Table 1). The human mesenchymal cell line (BM-MSC), was kindly provided by Toguchida Junya and Aoyama Tomoki at Kyoto University (Okamoto et al., 2002). A comparison between the no-stress SKP (NS-SKP), Tr-SKP and pH-SKP isolation methods was performed with normal dermal fibroblasts originally isolated from foreskin, as described previously (Wenzel et al., 2012b). All fibroblast cell lines were cultured as monocultures in DMEM (Sigma, D6429) supplemented with 15% fetal bovine serum (FBS, ThermoFisher-Gibco, 10270106), 1% L-glutamine (ThermoFisher-Gibco 25030081), 1% penicillin/streptomycin (ThermoFisher-Gibco, 1514022) and 0.4% gentamycin (ThermoFisher-Gibco, 15710049). Fibroblasts were subcultured and used when they were approximately 80% confluent because the use of confluent cultures resulted in poor SKP isolation and reduced viability.

All fibroblast cultures in this study were used at passage numbers ranging from 7 to 21. All cultures were performed in a cell incubator (Binder, 9140-0046) with a humidified chamber at 37°C and 5% CO_2_.

### Trypsin SKP isolation and culture

Trypsin-based isolation of SKP cells (Tr-SKP) was performed based on a previously described method (Wenzel et al., 2012b). Briefly, 80% confluent fibroblast cultures were washed with PBS and incubated in 5 ml of 0.25% trypsin-EDTA (ThermoFisher-Gibco, 25200056) for 16-18 h in a cell incubator at 37°C and 5% CO_2_. The cells were then pelleted (450×g, 5 min, room temperature) and washed in standard DMEM containing 15% FBS, followed by a PBS wash. One million cells were resuspended in 6 ml of classic SKP medium (Toma et al., 2005) [4:1-DMEM (ThermoFisher-Gibco, 21885025): F12 (ThermoFisher-Gibco, 21765029), 20 ng/ml EGF (ThermoFisher-Gibco, PHG0311), 40 ng/ml bFGF (ThermoFisher-Gibco, PHG0026), 2% v/v B27 (ThermoFisher-Gibco, 17504044), 0.5 µg/ml Fungizone (ThermoFisher-Gibco, 15290018) and 100 U/100 µg/ml penicillin/streptomycin] and equally divided over two T25 non-tissue culture treated flasks (Fisher Scientific-Falcon, 10112732). Cultures were fed every other day with 10× SKP medium (SKP medium with 10× concentrated EGF, bFGF and B27) diluted to a final concentration of 1× in culture media and agitated daily by pipetting up and down to prevent clumping or cell adherence to the plastic flask.

### Low pH SKP isolation and culture

Primary fibroblast cultures (80% confluent) were collected by trypsin and the cell suspension was pelleted at 450×***g*** for 5 min at RT, and washed with PBS. One million cells were resuspended in 500 µl of pH-adjusted HBSS (ThermoFisher-Gibco, 14175053) buffer. The pH of the HBSS buffer was adjusted with HCL (Merck, Hohenbrunn, Germany) to the following pH values: 7.0, 6.7, 6.3, 6.0, 5.7, 5.3, 5.0 and 2.5. Cells resuspended in HBSS at indicated pH were incubated for 25 min at 37°C and 5% CO_2_ and agitated every 5 min. Thereafter, the cell suspensions were centrifuged for 5 min (450×***g*** at RT). The cells were exposed to the indicated pH in HBSS for a total of 30 min, which included the 25-min incubation and 5-min centrifugation. Next, each pellet (containing 1 million cells) was resuspended in 6 ml of classic SKP medium and divided equally into two T25 non-treated culture flasks. The cultures were then maintained as described for the Tr-SKP condition.

### No stress SKP isolation and culture

Fibroblast cultures (80% confluent) were collected by trypsin, and the pellets were washed with DMEM containing 15% FBS, followed by two washes with PBS medium. Then one million cells were resuspended in 6 ml of SKP medium, equally divided into two T25 non-tissue-treated culture flasks and cultivated as described above.

### SKP spheroid analysis

Spheroid formation was determined by measuring individual spheroids via a whole-well oversight mosaic image. Spheroids from one flask were temporarily divided into two wells of a 48-well plate and placed in an Axiovert 200 M microscope chamber adjusted at 37°C and 5% CO_2_. A complete mosaic image was generated with the 5× objective for each well using the Zeiss Multidimensional Acquisition software (Axiovision 4.8.2). The captured image was cropped on the edges of the well and resized to 5000×5000 pixels in Photoshop CC (Adobe). The spheroids in the mosaic image were manually isolated with the Photoshop CC selection tool. Cellular objects smaller than 20 pixels were ignored. The spheroids were selected and colored in black, while the background was colored white, and the pictures were further processed in ImageJ (NIH). Each image was processed using the watershed tool and analyzed with the particle analysis tool (scale was set to 0.5 pixel=1.2 µm) to obtain the spheroid number and surface size of each individual spheroid per sample. The size frequency was calculated in Excel 2013 (Microsoft).

### Fluorescence-activated cell sorting flow cytometry

To determine cell viability, flow cytometry was performed via fluorescence-activated cell sorting (FACS). Cell viability was determined using the Count & Viability Kit (MCH100102; Merk Millipore, Germany) on the Muse Cell Analyzer (Merck Millipore) following the manufacturer's instructions. Data were analyzed and processed with Muse 1.4 Analysis software (Merck Millipore).

### Cryosectioning and fixation of pH-SKPs

The pH-SKP spheroids were cryosectioned prior to immunolabeling. Briefly, spheroids were collected and washed in PBS, and pelleted by centrifugation at 450×***g*** for 5 min at RT. The supernatant was removed and the spheroid pellet was resuspended in 30% sucrose (Sigma, 84097) in PBS. A 10- to 15-µl aliquot of spheroid suspension was embedded in optimal cutting temperature compound (OCT, Sakura, Finetek, Staufen, Germany, 4583) and fast frozen in a −80°C cooled 99% 2-propanol (Carl Roth, Karlsruhe, Germany, T910.1) bath. The pH-SKP spheroids were then sectioned at 5-µm thickness with a cryo-sectioner (Leica, CM3050S). Sections were fixed in ice-cold 100% methanol for 10 min at −20°C and then processed for immunohistochemistry.

### Immunohistochemistry

Cells grown on coverslips or slides containing spheroid sections were fixed with 4% paraformaldehyde (Merck, 1.04005) for 15 min at RT and then permeabilized with 0.3% Triton X-100 (Applichem, Darmstadt, Germany, A1388.0500) in PBS for 10 min at RT. Samples were blocked in PBS containing 15% FBS and 0.25% Tween-20 (Applichem, A4974.0500) for 1 h at RT. Next, samples were incubated with primary antibodies either for 1 h at RT or overnight at 4°C as indicated in Table S1. After several washes in blocking buffer, the corresponding secondary antibodies (Table S1) were added for 1 h at RT. Samples were counterstained and mounted with VectaShield containing 4′,6-diamidino-2-phenylindole (DAPI, Vector Laboratories–H1200).

### Differentiation of pH-SKPs

For all differentiation methods, pH-SKPs were first collected, placed in adherence medium (SKP medium supplemented with 5% FBS) and allowed to adhere in a 6-well plate for 16-18 h at 37°C and 5% CO_2_. BM-MSCs were similarly treated. For adipocyte differentiation, adhered pH-SKPs were washed once with PBS, and 3 ml of adipocyte differentiation medium {DMEM with 1 g/L glucose (ThermoFisher-Gibco, 21885025), 0.5 mM 3-isobutyl-1-methylxanthine [IBMX, Sigma, I7018, stock in absolute ethanol (VWR chemicals, 20821.33)], 10 µg/ml insulin [Sigma, I2643, stock in 0.01 M HCL (Merck, 1.00319.2500) in Ultra-Pure water from Milli Q (MQ)], 1 µM dexamethasone (Sigma, D4902, stock in absolute ethanol), 10% FBS, 0.5 µg/ml fungizone, 50 µM indomethacin [Sigma, I7378, stock in 100% DMSO (Sigma, D2650)] and 100 U/100 µg/ml penicillin/streptomycin} was added and completely refreshed every 2-3 days.

Oil Red O (ORO) staining following the ThermoScientific SC protocol 00011 confirmed adipocyte differentiation. ORO staining was observed by bright field microscopy, in which red staining indicated intracytoplasmic lipids.

For smooth muscle cell (SMC) differentiation, adhered spheroids (see adipocyte differentiation, adherence step) were dissociated by trypsin and seeded at a low density (2×10^4^ cells/well) in SKP medium supplemented with 5% FBS for 18 h. Fibroblasts and NS-SKPs were used in parallel. The cells were washed once with PBS and 3 ml of SMC differentiation medium [4:1–DMEM (Sigma, D6429): F12 and 2.5 ng/ml transforming growth factor beta (TGF-β1, ThermoFisher, PHG9214), 0.5% FBS and 100 U/100 µg/ml penicillin-streptomycin] was added to each well. The complete medium was changed every four days for a period of 8-12 days. SMC differentiation was confirmed by immunolabeling of the SMC markers calponin, αSMA and SM-MHC (Table S1).

For differentiation into fibroblast cells, pH-SKP spheroids were allowed to adhere for 18 h and split at low density as described for the SMC differentiation protocol. Cells were fed with standard DMEM/15% FBS fibroblast culture medium every 2 to 3 days. The cells were passaged 3 to 5 times before immunostaining for anti-prolyl-4-hydroxylase beta (P4HD), a fibroblast marker (Table S1).

Digital image analysis of ORO-stained adipocytes was performed to obtain a quantitative value of pH-SKP spheroids that had differentiated into adipocytes. Bright field images were acquired with a 10× objective providing a field of interest of 879 µm×658 µm. The images were processed and analyzed via a simplified method based on Deutsch et al. (2014). Briefly, via red color thresholding, cellular lipids stained by ORO were digitally isolated, followed by binarization of the image for digital analysis. ImageJ calculated the percentage of white color of the total image, which represented the percentage of adipocyte lipids. In total, three different image locations of the highest concentrated adipocyte areas per ORO-stained sample were analyzed. This procedure was performed with a total of three samples per cell line of interest to obtain a mean value with standard deviation.

### Statistical analyses

Results are presented as the mean±s.d. Comparisons were performed using Student's *t*-test. *P* values less than 0.05 were considered statistically significant. The sample sizes are indicated in the figure legends.

## Supplementary Material

Supplementary information
