# Supplementary material for: Rapid isolation and expansion of skin-derived precursor cells from human primary fibroblast cultures
Source: Biol Open. 2017 Nov 15;6(11):1745–55. doi: 10.1242/bio.025130 (PMC5703604; doi:10.1242/bio.025130)
Supplement: Supplementary information [file biolopen-6-025130-s1.pdf]

Supplementary information

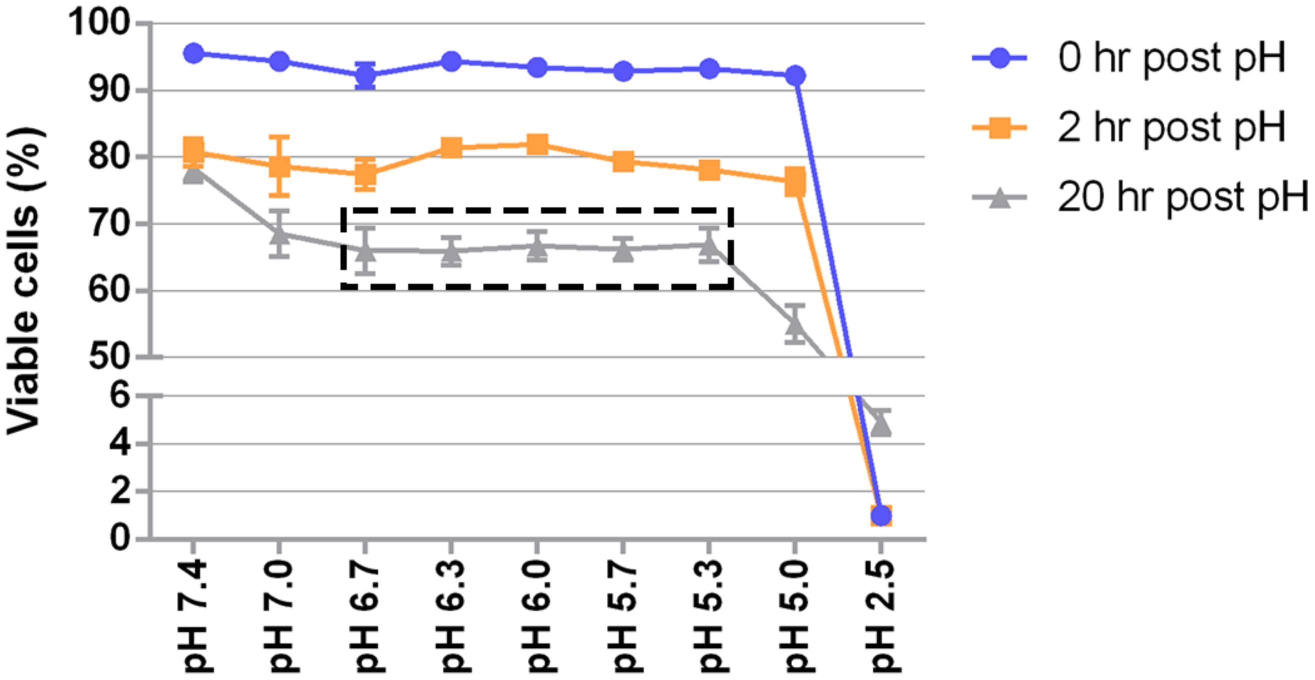

Figure S1. Primary dermal fibroblasts exposed to low pH from 6.7 to 5.3 exhibit a similar range of cell death. Viability assay graph based on fluorescence-activated cell sorting (FACS) analysis of foreskin primary fibroblasts treated in suspension for 30 minutes at 37°C with HBSS of different pH values as indicated. The percentages of viable cells at time points 0-, 2- and 20-hours post-treatment are shown as the mean ±S.D. (n=3).

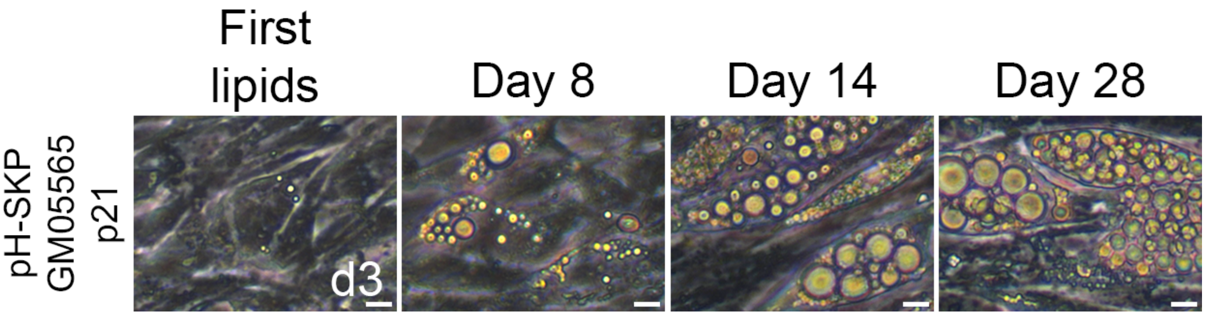

Figure S2. Adipocyte differentiation of pH-SKP spheroids isolated from primary fibroblasts at passage 21. Adipocyte differentiation of pH-SKP spheroids isolated from the cell line GM05565 at in vitro passage 21. The first column shows the day of first lipid detection at day 3, followed by days 7, 14 and 28 of adipocyte development. Scale bars: 10 µm.

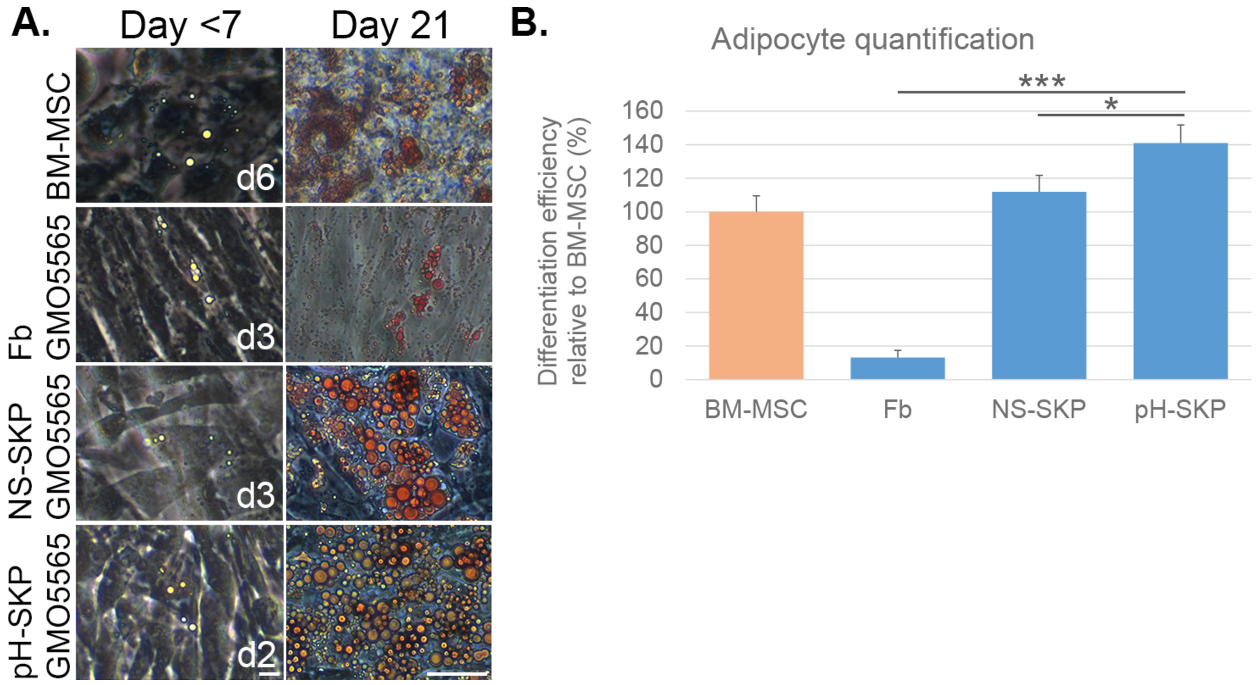

Figure S3. Adipocyte differentiation efficiency of pH-SKPs, NS-SKPs and fibroblasts. A.) Adipocyte differentiation of BM-MSCs, fibroblasts GM05565 (Fb), non-stress SKPs (NS-SKP) and low pH SKPs (pH-SKPs). The first column (Day<7) shows the first lipids detection, scale bar: 10 µm. Day 21 column shows Oil Red O (ORO) staining at day 28, scale bar: 50 µm (n=4). **B.)** ORO color threshold analysis at day 21 of adipocyte differentiation were performed with BM-MSC, GM05565 Fibroblasts (Fb), non-stress SKPs (NS-SKP) and acidic stress (pH-SKPs). Data are expressed as the mean ±S.D. relative to positive control BM-MSC (***p <0.0005, * p <0.05; n=4).

Biology Open • Supplementary information

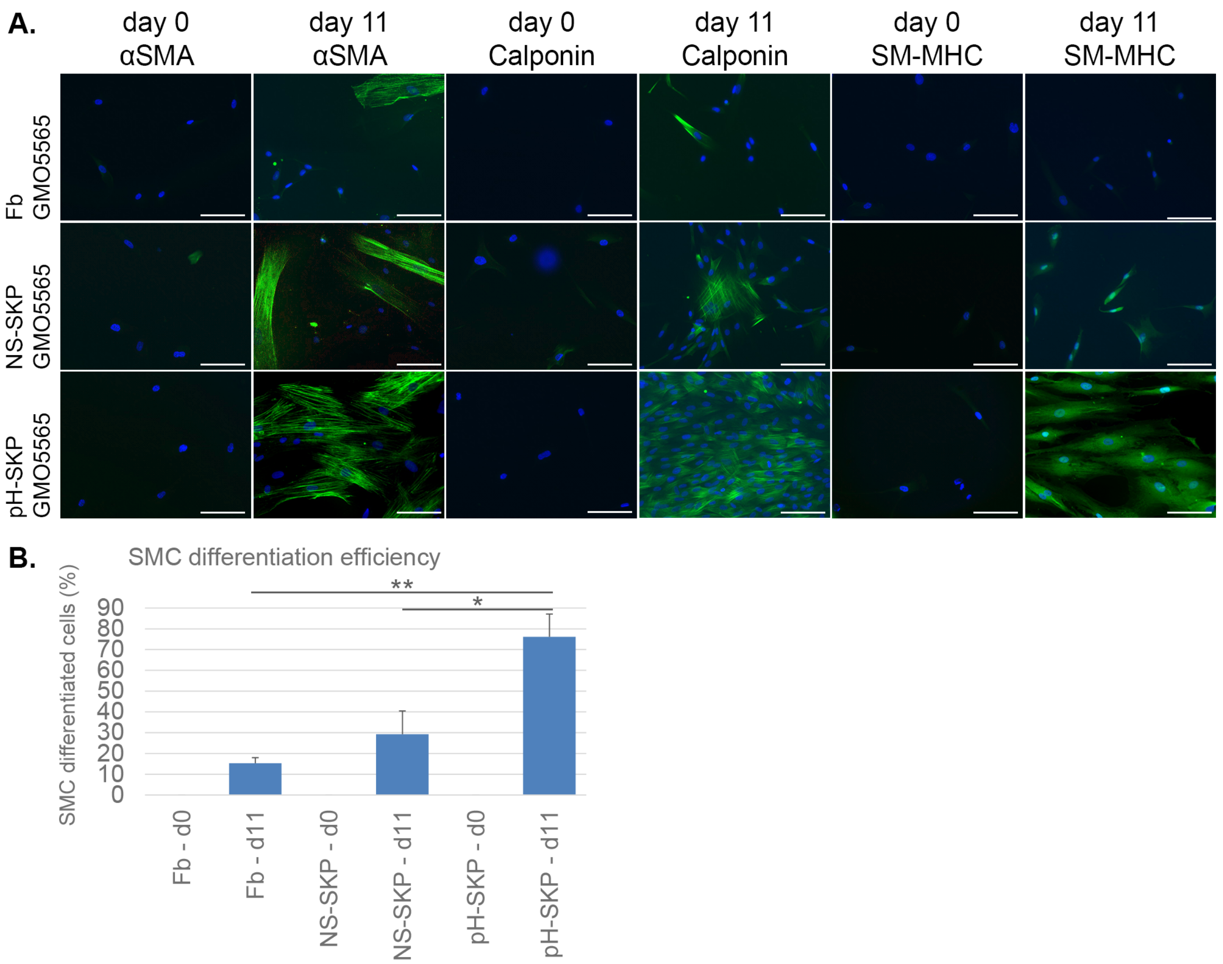

Figure S4. Smooth muscle cell differentiation efficiency of pH-SKPs, NS-SKPs and fibroblasts. A.) SMC differentiation of GM05565 Fibroblasts (Fb), non-stress SKPs (NS-SKP) and low pH SKPs (pH-SKPs) were monitored at day 0 and 11 by immunohistochemistry with antibodies for αSMA, Calponin and SM-MHC, detected in green. DNA was counterstained with DAPI (blue). Scale bar: 100 μm. **B.)** Quantification of SMC differentiation by determining the percentage of calponin-positive cells in indicated samples. The percentage was determined by direct count of at least 300 cells per coverslip in triplicate and from three independent experiments. Data are expressed as the mean ±S.D. (***p <0.0005, * p <0.05; n=3).

pH-SKP clonal assay

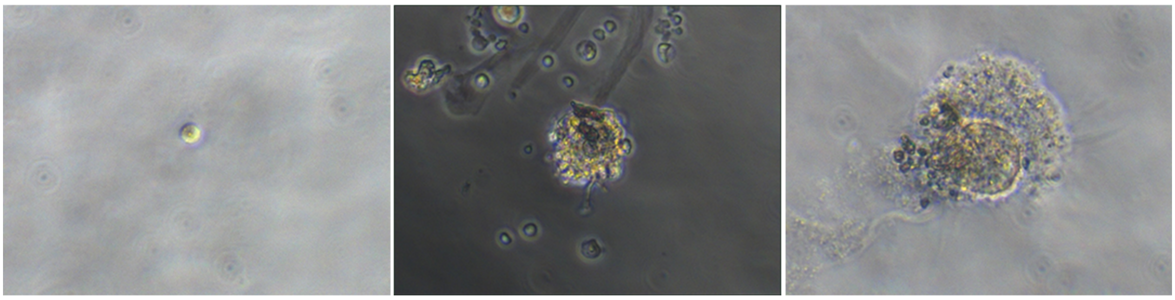

d0 d7 d14

Figure S5. Clonal growth of a single pH-SKP cell. A.) A single cell derived from GM05565 pH-SKP at day 5 via dissociation. The cell was followed for a total of 14 days.

Biology Open • Supplementary information

Table S1. List of antibodies used in immunohistochemistry.

**Primary antibodies**	**Fixation**	**Species**	**Dilution and incubation time**	**Catalogue no.**
**Stem cell markers**	Methanol			
anti-Nestin		Mouse	1:400 - 1 hr RT	Abcam, ab22035
anti-CD9		Mouse	1:400 - o/n 4°C	Millipore, MAB4427
anti-melanoma-associated chondroitin sulfate proteoglycan / neuron-glial antigen 2 (NG2/MCSP)		Mouse	1:200 - 1 hr RT	R&D, MAB2585
anti-Vimentin		Mouse	1:200 - o/n 4°C	Millipore, CBL202
**Smooth Muscle Cell markers**	PFA			
anti-Calponin		Mouse	1:100 - o/n 4°C	Dako, M3556
anti-α-smooth muscle actin (αSMA)		Mouse	1:100 - o/n 4°C	Dako, M0851
anti-smooth muscle myosin heavy chain (SM-MHC)		Mouse	1:200 - o/n 4°C	Abcam, Ab683
**Fibroblast marker**	PFA			
anti-prolyl-4-hydroxylase beta (P4HD)		Mouse	1:400 - o/n 4°C	Acrys, AF0910-1
**Other**	Methanol			
anti-Fibronectin		Rabbit	1:400 - 1 hr RT	Sigma, F3648
anti-Lamin A		Mouse	1:200 - 1 hr RT	Abcam, ab8980
**Secondary antibodies**	-			
anti-Mouse IgG (H+L)		Donkey	1:400 - 1 hr RT	ThermoFisher, A-21202
anti-Rabbit IgG (H+L)		Donkey	1:800 - 1 hr RT	ThermoFisher, A-31572

Biology Open • Supplementary information
